# ‘Come on, Give Me the Pills Now’: A Narrative Analysis of Reproductive Agency in Self‐Managed Abortion in Argentina

**DOI:** 10.1111/1467-9566.70181

**Published:** 2026-04-08

**Authors:** Mercedes Vila Ortiz, Fiorella Guaglianone, Antonella Lavelanet, Vanessa Brizuela, Anna Thorson, Anna Kågesten, Mariana Romero, Amanda Cleeve

**Affiliations:** ^1^ Department of Global Public Health Karolinska Institutet Solna Sweden; ^2^ Centro Rosarino de Estudios Perinatales Rosario Argentina; ^3^ Universidad Nacional de Entre Ríos Paraná Argentina; ^4^ Consejo Nacional de Investigaciones Científicas y Técnicas (CONICET) Buenos Aires Argentina; ^5^ Department of Sexual and Reproductive Health and Research UNDP‐UNFPA‐UNICEF‐WHO‐World Bank Special Programme of Research Development and Research Training in Human Reproduction (HRP) World Health Organization Geneva Switzerland; ^6^ Special Programme for Research & Training in Tropical Diseases World Health Organization Geneva Switzerland; ^7^ Centro de Estudios de Estado y Sociedad Buenos Aires Argentina; ^8^ Department of Women's and Children's Health Karolinska Institutet Solna Sweden; ^9^ WHO Collaborating Center for Human Reproduction Karolinska University Hospital Solna Sweden

## Abstract

Recent discussions on self‐managed abortion highlight its potential to reduce risks and delays while supporting reproductive rights. This qualitative paper analyses the stories of 12 women who self‐managed their abortions in Argentina. In‐depth interviews were conducted in April and May 2024. Using a framework of reproductive agency, the paper explores how legal, social, economic and personal circumstances shaped self‐management experiences. We conducted a narrative analysis, resulting in four narrative types: self‐managed abortion as empowerment, as navigating the unexpected, as moral hesitation and perceived health risk, and as a private experience. Some participants described their experience as empowering, while others expressed ambivalence, fear and concern about health risks while exercising reproductive choice. Access to sexual and reproductive health information emerged as a key enabler of agency, though it was unevenly distributed, reflecting broader structural inequalities. Emotional and physical challenges were common, even among those confident in their decision, highlighting the need for care models that acknowledge the complexity of abortion practices. The findings call for a person‐centred approach that support diverse pathways to abortion care. Such flexibility is essential to advancing reproductive agency and equity.

## 1 Introduction

Self‐managed abortion (SMA) usually refers to an abortion that an individual self‐manages using pills, either in part or fully, without direct supervision from a health worker (World Health Organization 2022; Berer 2020). In the last decades, discussions on SMA have increasingly focused on how this practice may contribute to reducing risks and avoiding delays while enabling the realisation of reproductive rights (Jacobson and Gerdts 2025). SMA can increase abortion access and expand reproductive choice, allowing pregnant individuals to realise their preferences and values (Erdman et al. 2018). However, experiences of SMA differ and are closely tied to the circumstances in which they take place, such as the availability of healthcare services, support networks, access to accurate sexual and reproductive health and rights (SRHR) information and the legal and social environment (Kristianingrum et al. 2022; Atienzo et al. 2023). In settings where abortion is legal, the option of self‐management may be incorporated within formal healthcare service delivery. Nevertheless, women may still opt to self‐manage their abortion outside the formal healthcare system due to access barriers or because they value the confidentiality and privacy that self‐care practices offer (Stapff et al. 2025). In contexts where abortion is legally restricted and/or highly stigmatised, SMA is a critical avenue for people who wish to end their pregnancy (Zamberlin et al. 2012). In these settings, SMA is often performed clandestinely (Bury et al. 2012; Madeiro and Diniz 2015).

In Argentina, abortion has been legal on request up to 14 weeks of pregnancy since 2021. Since before the legalisation, misoprostol has been widely used for SMA, often obtained through pharmacies—with or without a prescription, despite the legal restrictions in place—or through informal networks and online sources (Zamberlin et al. 2012). Feminist collectives have played a crucial role in supporting SMA throughout the country before and after abortion was legalised (Luchetti et al. 2024; Burton and Peralta 2021). Before the abortion law, these groups provided information and accompaniment to abortion seekers, including instructions on how to safely use abortion medications and access healthcare services, even though their work operated in a legally grey area and carried risks of criminalisation (Zurbriggen et al. 2018). SMA with the support of feminist organisations has been an important avenue for individuals facing barriers to legal abortion in the country, offering a safe alternative outside healthcare settings in a context where abortion was largely criminalised (Keefe‐Oates et al. 2022). Moreover, research shows that SMA with support from accompaniment groups is safe and effective, with outcomes similar to clinician‐provided care, especially in early pregnancy (Moseson et al. 2022). Feminist collectives have continued to support SMA after the 2020 legislation, as the demand has persisted, both to accompany abortions outside the healthcare system or to facilitate access to the healthcare system, creating a continuum between self‐managed and clinical care (Keefe‐Oates et al. 2025; Vila Ortiz et al. 2024). In parallel, the number of healthcare facilities offering abortion services, including the provision of pills for home use, has doubled by 2022, two years following legalisation (Romero et al. 2024).

In this paper, we analyse 12 testimonies of cisgender women who self‐managed their abortion between 2001 and 2021, including how legal, social, personal, and practical circumstances shaped their experiences and needs during the process. Previous research studies on the topic in Argentina have focused on the experiences of people who have self‐managed with the accompaniment of feminist collectives (Luchetti et al. 2024; Keefe‐Oates et al. 2025). These studies have explored the reasons behind choosing this type of support, while others have focused on the barriers abortion seekers encounter when accessing abortion services in the healthcare system (Keefe‐Oates et al. 2022; Szulik and Zamberlin 2020; Tiseyra et al. 2022). Building on these findings, we were interested in exploring the experiences of people who self‐managed without the support of the formal healthcare systems during different legal and social contexts in Argentina. We focused on how women narrated and made sense of their SMA experience as part of their life trajectories and how they exercised *reproductive agency* within their personal circumstances and contexts. By delving into the experiential aspects of SMA, we hope to inform health systems on how to better meet the needs and preferences of those who self‐manage.

## 2 Theoretical Framework: Shaping Reproductive Agency

This study draws on theories that conceptualise agency not as a purely individual capacity but as a context‐dependent practice shaped by power structures, subjectivation processes and collective action (Ortner 2006; Rebughini 2023). We defined and operationalised the concept of reproductive agency using the Conceptual Framework for Reproductive Empowerment developed by the International Centre for Research on Women (Edmeades et al. 2018). This framework has been previously used in a recent study that analysed agency in the decision‐making processes related to maternal health care‐seeking (Olwanda et al. 2024). In our study, we applied the framework to SMA because it captures reproductive agency as a multidimensional and context‐dependent process, which is particularly relevant where abortion experiences involve navigating legal restrictions, social stigma, and uneven access to health systems (Hyatt et al. 2022). This framework defines agency as ‘the capacity for purposive action that draws on social and material resources at multiple levels to realise preferences and choices, enhance voice, and increase power and influence’ (Edmeades et al. 2018). It identifies three dimensions of agency: *choice*, the capacity of people to make decisions regarding their own life and health; *voice*, the ability to express intentions, desires, preferences as well as demand change, participation in decision‐making, etc., and *power*, understood in its various dimensions of dominance, influence, resistance, etc. (Edmeades et al. 2018), including the capacity to act created and enabled by specific relations of subordination (Cense 2019). This definition emphasises the different circumstances that *shape* the exercise of agency. This includes individual factors (e.g., educational status and knowledge of SRHR), relational factors (e.g., support or coercion of family, partners and people providing healthcare) and structural factors (e.g., people's position in the social structure, the availability and accessibility of SRHR information and medication, the legal context, abortion policies, gender norms, social expectations, etc.) (Edmeades et al. 2018).

## 3 Methods

### 3.1 Study Design

This qualitative study was carried out within the context of a large cross‐sectional study on SMA conducted in Argentina. Recruitment for the qualitative interviews followed a web‐based Respondent Driven Sampling (webRDS) survey which was conducted between February and May 2024, with results presented in other papers (Cleeve et al. 2026). In short, survey participants were individuals who had ever been pregnant, who were between the ages of 16 and 49 and resided in Argentina at the time of the survey. Between April and May 2024, in‐depth interviews were conducted among a subsample of survey participants who had reported experiencing SMA outside the formal health system in the country. We defined SMA outside the formal healthcare system as having obtaining abortion pills without visiting or contacting a health facility and self‐administering the pills at home, regardless of whether they received support from a community accompaniment group. Seeking pre or post‐abortion care was not part of the exclusion criteria. This qualitative study reports the results of the in‐depth interviews.

### 3.2 Setting

Argentina has progressively adopted a rights‐based approach to SRHR (Romero and Ramón Michel 2022). Abortion was historically criminalised, with exceptions of risk to life or health, and rape, and access was often hindered by judicial and institutional barriers (Ramón Michel and Ariza Navarrete 2018). A turning point came in 2012 with the Supreme Court's F.A.L. ruling (National Supreme Court of Justice of Argentina 2012), which removed the need for judicial approval for rape cases, applied the rape ground to all women (not just those with mental disabilities) and expanded the ‘health’ ground to include mental and social well‐being. Following this, national and provincial protocols sought to operationalise access, though implementation varied (Ramón Michel and Ariza Navarrete 2018). Strong feminist advocacy across the country culminated in Law 27.610 (Congreso de la Nación Argentina 2021), legalising abortion on request up to 14 weeks and under grounds thereafter, though implementation challenges remain (Romero et al. 2024; Ramos et al. 2023).

### 3.3 Reflexivity Statement

We engaged in continuous reflexivity throughout this study. We are an interdisciplinary team, comprising cisgender women working in diverse institutional settings across high and middle‐income countries. We acknowledge that our positionalities as researchers embedded in SRHR shaped the research process. Rather than viewing this as a source of bias, we embraced our subjectivity as a resource for constructing knowledge with participants (Olmos‐Vega et al. 2022). The diversity of our backgrounds, some rooted in the SRHR advocacy field and others not, provided multiple vantage points, fostering a more nuanced understanding of the data. At the same time, we remained critically engaged with the data and actively interrogated dominant and alternative framings of SMA. Our reflexive approach involved examining how our experiences and assumptions might influence our interpretations, while also creating space for alternative perspectives to emerge. For example, when developing the narrative of *SMA as empowerment* and *moral hesitation*, we critically interrogated whether the advocacy background of some of the researchers in the team might predispose us to emphasise empowerment over ambivalence or moral dilemmas. Team‐based discussions also helped us to be mindful of less common perspectives, such as narratives that critically reflected upon accompaniment practices or highlighted physical pain of the person having the SMA. Moreover, we held discussions to reflect on power dynamics between researchers and participants, particularly given the sensitivity of the topic.

### 3.4 Recruitment and Data Collection

Participants in the webRDS survey who indicated that they had a SMA experience outside the formal healthcare system were asked about their willingness to participate in a follow‐up in‐depth interview. Those who agreed were asked to provide an email address. Nineteen survey participants were sent an email containing information about the follow‐up interview and the informed consent document. Twelve responded and agreed to participate in the interview. After informed consent, women were interviewed in Spanish on video conference or phone, depending on their preference. We used a semi‐structured guide with open‐ended questions and probes. The interview guide is available in Annex 1.

The interviews took place at a time that was convenient for each participant and lasted between 26 and 62 min. All the interviews were conducted by MVO, a social scientist working in SRHR research, with previous experience interviewing people about their abortion experiences. All interviews were audio recorded and transcribed verbatim. Audio files were kept separate from pseudonymised transcribed data in an encrypted server. They were analysed in Spanish, and only the selected quotes were translated into English. Although we had set out to interview individuals who indicated that they had got the pills and carried out the abortion outside the formal healthcare system, during the interviews, we found out that three participants had obtained pills from a healthcare provider in a healthcare facility. We considered that their narratives still significantly contributed to our understanding of the SMA experience and our study aim and, therefore, included them in the analysis.

### 3.5 Participant's Characteristics

**FIGURE 1:**
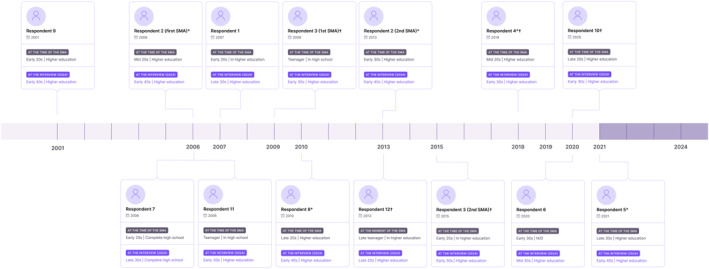
Time of the abortion(s), age and educational level. *SMA supported by a community accompaniment group. † Pills were obtained from a provider in a health facility.

Twelve cis women from four different jurisdictions of the country (Buenos Aires province *n* = 3, Buenos Aires city *n* = 4, Entre Ríos province *n* = 3, and Santa Fe province *n* = 2) participated in the study. Twelve participants narrated the experience of 14 abortions. Figure 1 shows the time at which the abortion(s) took place, the age (using broad bands) and educational level of participants at the time of the SMA and at the time of the interview, and whether they received community accompaniment or obtained the pills in the healthcare system. Most participants underwent their first or only SMA when abortion was still criminalised (*n* = 8), others did so during a period of greater social and institutional acceptance of the practice, when national and several provincial abortion protocols were in place (*n* = 3) and one after full legalisation (*n* = 1).

### 3.6 Data Analysis

We analysed participants' interviews using a narrative approach. Narrative inquiry looks at the way stories are built, based on the premise that one way people use to create and attribute meaning(s) to their experiences is telling stories (Riessman 1993; Clandinin and Connelly 2000). We chose this approach as it allowed us to keep the experiential aspect of each story, contextualising meaning making (Riley and Hawe 2005), while achieving a higher level of abstraction.

Two researchers, MVO and FG, carried out the analysis with the help of AC and MR. Analysis followed two stages: (1) Mapping narratives in a matrix according to our theoretical framework and (2) identifying narrative patterns to build four different narrative types.

At the beginning of the first stage, we read the interview transcripts to familiarise ourselves with the narrations and identify the main features of each story. We then mapped each interview in an Excel matrix using both an inductive and deductive approach. We inductively extracted information on several characteristics regarding the content of each story. Other characteristics were mapped deductively. Following our theoretical framework, we looked at how agency was exercised, including the dimensions of choice, voice and power. With the same framework, we analysed the personal, relational and structural circumstances that shaped the exercise of agency. Finally, we analysed the three key dimensions for narrative inquiry, according to Clandinin and Connelly (2000): temporality, sociality and spatiality. Details on the mapping process is available in Annex 2.

In a second stage, we identified narrative patterns in how participants made sense of their SMA experience and how they positioned themselves in relation to agency. We engaged in iterative discussions with the rest of the co‐authors, using the matrix described above to compare the different narratives. These discussions led to the development of four narrative types. Rather than assigning participants to a single narrative, we built these types with different aspects emerging from the interviews, acknowledging that individual stories often contained elements of more than one narrative. Each type is organised around central concepts that describe the most salient features of how the SMA was narrated and made sense of. These narrative types were constructed by conceptually overlaying the following guiding questions onto the framework: *How did participants narrate their SMA experiences as part of their reproductive life trajectory? How did participants position themselves in relation to agency when narrating their SMA experience?*

### 3.7 Ethics Statement

The project received ethical approvals from the WHO Ethics Review Commission [A66017], Investigación y Reflexión Bioética (www.irbioetica.com) and RESPIRE, Argentina. Participants were asked to provide electronic consent, followed by verbal consent, before the recording of the interview began. We informed them that their participation was voluntary and confidential and that they could skip any question and/or end the interview at any stage without consequences.

## 4 Results

We built four types of narratives: SMA as empowerment, SMA as navigating the unexpected, SMA as moral hesitation and perceived health risk, and SMA as a private experience.

### 4.1 Narratives of SMA as Empowerment

Across several interviews, SMA often was narrated as an *empowering process*: an opportunity to make autonomous decisions and build self‐assurance. Empowerment appeared in both individual and collective forms. Some participants emphasised individual agency and self‐confidence, describing how they managed to obtain medication outside the health system with scarce information on SRHR, facing fear and anxiety at a time when abortion was illegal. For instance, one participant narrated:

At that time I think I was vulnerable regarding these issues and I feel that in spite of everything I was able to handle that situation (…) What I felt was the exposure, the issue of what others would think, the taboo around this subject at that time (…) When I think about this experience I feel relief and I feel empowerment. Saying “I was able”. (…) I guess I also trusted my judgement because I did a whole day of research on the Internet to see which was the option that seemed the most reliable and safe. (…) As an ideal situation… it is a bit closer to the situation we have now, where abortion is no longer a taboo (…) I feel like it’s something that’s discussed much more naturally now. (…)

(Participant one, SMA in 2007)

This same quote also reveals how empowerment coexisted with, and ultimately overcame, feelings of exposure and taboo, overlapping with the narrative of *moral hesitation*. The process of overcoming vulnerability through purposive action highlights the dimension of power within agency, as the participant positioned herself as capable of acting despite social constraints. Similarly, participant 12 narrated having searched the Internet for information about the SMA process and feeling empowered when she was able to make autonomous decisions against conservative family values and to self‐manage with limited knowledge and support:

I think it [the SMA] marked a way of thinking in which I felt responsible for my decision and knew that I was capable of deciding and taking the path I wanted (…) I had the power to decide, to say: “Well, I don't want this for my life, I'm not going to do it this way, I'm not going to have a child”

(Participant twelve, SMA in 2013)

These participants believed that informing themselves about SRH was the most effective way of dealing with their feelings of anxiety and fear during the SMA. The process of learning and independent decision‐making was regarded as an important part of exercising agency. In their view, the most difficult parts of SMA could be more easily dealt with if people could openly discuss SRH matters. In other interviews, empowerment was narrated as a collective endeavour, highlighting the crucial role of feminist activism in facilitating SMA and making it an agentive experience. For instance, participant two, who had her first abortion by obtaining pills through an international women's organisation online at a time when abortion was illegal, found that becoming an activist in the feminist movement locally helped her re‐signify the SMA:

I was upset for a long time… I counted the months, “oh, it would have been born at this time, this date would have been its birthday, it would have been this zodiac sign”. Then, well, there was a whole process in my life that took away the guilt, that I was able to process all that, that I became an activist in feminism and all that. The second time [I had an abortion] (…) I had a different perspective.

(Participant two, SMA in 2006 and 2013)

SMA was narrated an opportunity to exercise agency within an activist framework in several interviews. Feminism provided a collective way of interpreting, experiencing and sharing their SMA, allowing participants to voice their preferences in safe spaces and contrast them with dominant patriarchal and religious values. Several participants highlighted the support they received from feminist accompaniment groups who support SMA in Argentina. This was clearly expressed, for example, by participant four, who, after being accompanied during her SMA, became a companion herself, in a context of increasing social acceptance of abortion:

I felt supported, yes, I felt supported by the ladies [from the accompaniment group] (…) who made it possible for me to get the medication (…). And I felt supported by my friend, and I felt supported (…) by the whole Green Tide,^1^ which also helped me reach the decision in a safe way, as my own personal process within feminism and activism. (…) I remember that [my] first [SMA] accompaniment (…) It was very beautiful. And what is also beautiful about [accompaniment groups] is that it is like a constant training, like this thing of generating knowledge from the abortions of those we accompany.

(Participant four, SMA in 2018)

In this narrative type, having information about SRH and advocating for change in how abortion is considered at a social level (taboo vs. social acceptance and openness to talk about abortion) were deemed essential to have an empowering experience of SMA. In this line, reproductive agency was linked to having the possibility of making informed and supported decisions from a SRHR and feminist perspective, either from a collective or an individual stance, as pointed out by participant five:

If you ask me why I am part of [feminist group advocating for abortion rights], part of it has to do with my own paths, and another with the emancipatory project (…). I think there is a deliberate decision by patriarchal, capitalist, racist, colonial systems for us to be reproductive bodies, and I see [ours] as a project of autonomy, decision‐making, and freedom. For me, it is part of a social transformation project. For others, it is more about individual freedom (…). My reasons for advocating were not personal, singular, individual. Clearly, with the abortion, they did become personal. They [personal reasons] were added.

(Participant five, SMA in 2021)

It is worth noting that the participants whose stories contributed to the *empowerment* narrative had completed or were pursuing higher education at the time of the abortion, with high literacy levels and internet access, circumstances that likely facilitated their ability to seek information and/or feminist accompaniment groups and exercise reproductive choice.

### 4.2 Narratives of SMA as Navigating the Unexpected

Another narrative which appeared in some interviews was around the experience of the unexpected. For example, participants five and eight described their SMA as a more complex experience than they had expected it to be. Because they both were activists in different political organisations at the time of their SMA, they considered themselves fitted with the personal and relational resources required to make autonomous decisions and handle, relatively easily, the SMA process. This sense of preparedness and autonomy also resonates with elements of the *empowerment* narrative. However, their experience was narrated as not being as simple as they had anticipated, with different circumstances shaping their path and their position in relation to agency.

Participant eight highlighted that, at the time she had the SMA in 2010, feminist organisations had just begun to provide support for doing abortions with pills at home in a context in which abortion was illegal. Coming from an activist background, she contacted a feminist accompaniment organisation, which supported her throughout the process. She felt completely certain about her decision and had the necessary information and connections to self‐manage an abortion. Still, she was taken aback by the apprehension she felt due to the illegality of the practice and the uncertainty of performing a SMA without medical support. She feared dying or being sent to prison and expressed that the dominant social discourses around SMA at the time prevented her from having what we conceptualise as an agentive experience. She hypothesised that if the social context had not been criminalising, the SMA would have been easier to go through:

The issue is the power of illegality, even when you know that what you are doing is right, because I had no doubts about it, neither morally nor ethically or anything else (…) Yes, I was afraid of dying. I was really scared to death, because of the context in which I was doing it, in the most absolute illegality. And then death in terms of health, in other words… what the fuck am I doing? Not because I doubted what I was doing, but because one is not a doctor. (…) But well, I think it's much easier to say it today. And if you can say it more easily, you can go through it more easily (…).

(Participant eight, SMA in 2010)

Although this account intersects with the *empowerment* narrative by displaying confidence in the decision to have an abortion, it highlights how criminalisation of the practice shaped and constrained agency by causing fear and uncertainty. A somewhat similar situation was narrated by participant five, who had her SMA when abortion had just been legalised, and was herself an abortion activist in a group advocating for abortion rights. She chose a SMA because she had close connections to feminist groups that accompanied abortions. She positioned herself as someone who knew and supported SRHR before having had an abortion herself. During her own SMA, she narrated having become aware of the ‘materiality of the body’ of the person aborting, in particular the physical pain she felt during the abortion. Her (self‐)critique was directed at the way feminist activism sometimes handled this dimension of physicality in the accompaniment of SMA:

There was something there about the body… a dimension that I did not consider before. I had considered the political [dimension], the symbolic cultural one (…), but well, not the singular one. I had accompanied a lot of terminations of pregnancy [outside the health system], but I had never taken charge of this. (…) And now after, I specifically talk about this, beyond the decision, there is going to be a body that is put at stake, that is going to hurt. (…) I hear the rest of my activist colleagues, and I notice that this is still not mentioned

(Participant five, SMA in 2021)

Although both participants criticised the existing social and patriarchal discourses around abortion, there was a key difference between them in what was desirable regarding agency when self‐managing. Participant eight expressed that the social and legal context shaped the nature of the SMA experience in terms of its social acceptability and the availability of support from the formal health system. Participant five, on the other hand, felt that there is something inherently beyond one's control in the SMA experience—an ontological dimension of insecurity (Lorey 2015)—that persists even in settings where social values are supportive and formal support systems are in place. In her account, the pain and fear associated with SMA were not solely products of legal or social conditions but also stemmed from deeper and, from her perspective, unavoidable uncertainties.

### 4.3 Narratives of SMA as Moral Hesitation and Perceived Health Risk

Several interview excerpts highlighted the moral dimension of abortion, often intertwined with concerns about health risks, particularly in the absence of medical supervision. For many participants, the belief that abortion was morally wrong intensified fears of health consequences, such as infertility or severe complications in future pregnancies. SMA was portrayed both as a last resort to exercise reproductive choice and as an insecure practice. Medical knowledge was consistently valued as a source of safety, yet often inaccessible. Importantly, this narrative was most evident among participants with limited economic resources, little information on SRHR and who obtained pills outside the formal healthcare system with little or no support. These conditions significantly constrained reproductive agency. For example, participant three, who had two abortions when the practice was still criminalised, focused her SMA narrative on the guilt of ‘having killed a human being’ and fear of physical consequences of abortion:

Look, it [the abortion] caused a problem in my body, because after this, I don’t know why, since I’ve had millions of tests and nothing shows up, but I can’t get pregnant (…).Not everyone is indeed affected, or maybe it doesn’t affect you to kill a human being, because you are killing a human being (…) I think it does influence you.

(Participant three, SMA in 2009 and 2015)

This participant also expressed concern about the dangers of performing an SMA without medical supervision, noting that her abortion occurred within a conservative family setting, where she lacked access to SRH information and parental support. She also highlighted the risks of young teenagers attempting the procedure without medical guidance. In her interview, she emphasised that she would have liked to receive professional healthcare:

If I could go back, I don't know, I would have liked to do everything I did with the help of a professional, because I think doing it, for example, within a medical institution would make you feel safer, more at ease if something happens (…) Also, because of the quality of the pills or whatever might affect you, because I don't know what they might sell you (…) [When my daughter] is 12, 13 years old, if something like that happens to her and without knowing I come back and find her bleeding to death in bed… It's wrong that it's so easy to have access to this type of medication.

(Participant three, SMA in 2009 and 2015)

Similarly, another excerpt from participant 11 emphasised her concerns about potential health consequences following her SMA, interwoven with the social taboo around abortion and misinformation around the practice:

But I clearly remember that [the doctor] told me I couldn't get pregnant for two years because the baby would have malformations, be sick, or have some defect. I remember that very clearly because I got pregnant again three months later and was terrified (…) I was very ashamed. At that time, I should have seen a psychologist or someone who could support me more. I was with my friend, who was also 15 years old

(Participant eleven, SMA in 2006)

Faced with limited options due to relational factors (lack of support and accompaniment and relying only on a 15‐year‐old friend) and structural constraints (limited economic and social resources to easily access pills and lack of comprehensive information on how to use the pills), SMA in this narrative was depicted as a useful yet risky option. For many participants, SMA was far from being an empowering experience and reproductive choice was exercised in a context of constraint. For example, participant 11 was exposed to different types of violence while pursuing self‐management. These included being left alone during the process by her parents, denied detailed information by a pharmacist, mistreated when seeking help at a health facility and subjected to misinformation and scaremongering by healthcare providers. Even when she was able to act, power was exerted from a position of resistance:

I got pregnant when I was 15. (…) My mom advised me to buy a pill. We didn't know the name (…). I went to the pharmacy. I wrote the name on a piece of paper, went to the pharmacy alone, and bought the pill. At that time, it was very expensive (…) The pharmacist asked me if I knew what it was, and I said yes. “Do you know how to use it?” he asked, somewhat arrogantly (…) I said no. And he said, “Well, you take two, you take two,” he said, (…) I didn't have much more information. So I went home, followed the pharmacist's instructions. By the evening, I was passing huge blood clots. I had never seen something so big come out of me. And I got scared, I got scared, alone in my house

(Participant eleven, SMA in 2006)

Participant nine's account also emphasised the connection between moral ambivalence and fear of health consequences of a SMA. This participant obtained the pills through the black market and had an SMA clandestinely in a criminalising context, which could explain her heightened sense of risk. At the same time, she expressed a positive view of healthcare oversight as a source of safety:

I got the pills by word of mouth, there’s a lady who gets you the pills, she worked in healthcare. She sold them to me (…) The thing is, I have a constant inner conflict about the issue because, while I believe everyone has the right to choose, I also think we need to work on the prevention because it’s not something pleasant to go through, it’s not like having a sore throat and taking a pill. So, whatever the method, it is unnatural, and whether you like it or not, health‐wise it leaves consequences, some harm (…) That was my fear. This came up and I thought: what if one day I can’t have children? Nowadays [the abortion] would be in a safe setting, medically supervised and everything else. So, if it’s done with the utmost care, it shouldn’t have an impact, right? But when you do it clandestinely…

(Participant nine, SMA in 2001)

### 4.4 Narratives of SMA as a Private Experience

Across some interviews, SMA was narrated as a deeply private experience, emphasising the uniqueness and interiority of the process. In this narrative type, having the necessary space to navigate the experience without being forced to talk about it outside their close sphere, that is, close friends and family, was highly valued.

For example, participant six had her abortion in 2020, right before the legalisation of abortion. She purchased the pills from an online source after contacting both the health system and a feminist accompaniment group. Although she approached places that were friendly in terms of abortion access, she criticised the waiting times she faced. She perceived that the steps required to access abortion, such as consulting a psychologist at the health centre or attending a workshop at the accompaniment group, were cumbersome and bureaucratic, particularly due to the lack of clarity on whether and when she would get the pills. Feeling anxious and unsure about whether she would succeed in terminating her pregnancy, she reported that she just wanted to get things done:

I went to one of those health centres (…) to see if they could give me the medication and no, they gave me an appointment with a psychologist, I don't know, (…) and I felt that they were going round in circles, so I kept looking for other alternatives. [Then she contacted a feminist accompaniment group] I don't know what they wanted to do, to get together to debate, (…), to propose a dialogue, to chat. And I thought: what's up? It is the same as the other, but different. I mean, I don't understand. Come on, give me the pills now

(Participant six, SMA in 2020)

For her, SMA was something she should have been able to deal with straightforwardly and with the support of people close to her and whom she chose herself. In this context, the requirements of the healthcare system—imposed in the face of what she experienced as an urgent need—felt like an infringement, a loss of what we have conceptualised as agency. She described SMA as a good option to navigate an intimate experience and acknowledged that prior consultation with health services (including the psychologist) is useful as long as it is quick and straightforward:

And yes, the truth is, obviously, if you feel supported, if you are in a place with your mom or your family, if you are sure of what you are doing, previously going to the doctor, if possible to the psychologist (…) I mean, I would prefer to do it. Besides, it's something so intimate, that it has the good side of doing it. I would recommend it, of course, but with prior counselling. (…) And I would have liked, when I went to the health centre for the first time, if they had advised me and been more expeditious in saying: 'Yes, we will give you the pills, don't worry’

(Participant six, SMA in 2019)

Participant seven’s interview also contributed to this narrative. Coming from a desire to avoid the powerlessness she felt when she was not able to decide whether to interrupt her first pregnancy when she was 15 years‐old, she decided to self‐manage. With pills provided by a pharmacist she was acquainted with but without much information on how to use them, she recalled having no sense of control and stressed that what matters most is the private dimension of the experience and the support of close friends:

I didn't expect to have control over anything. I was more like, well, take this pill, take this pill and that's it, and see what happens. (…) But there is nothing philosophical or ideological there that changed before or after. (…) Yes, I would have liked to have support, as I said, recently we went through it with a friend. And she said, ‘just having you girls there to accompany me’, I felt that what she was experiencing was not the same as what I went through. (…)

(Participant seven, SMA in 2006)

## 5 Discussion

In this paper, we explored how 12 women narrated their experiences of SMA, with particular attention to how they positioned themselves in terms of agency. Our findings revealed that SMA was made sense of in diverse ways: although SMA was sometimes described as empowering, other times it was expressed as a moral conflict or ambivalence, even when it appeared as the only option to exercise reproductive choice. The narratives highlighted that agency emerged in multiple ways. In some accounts, agency was explicitly articulated as decisive action and control, whereas in others, it was more implicit or constrained and not always recognised as such by the participants themselves. In general, participants with higher education, economic resources, digital literacy and/or contact with activist networks tended to explicitly narrate their experience as exercising agency more fully, whereas those facing the compounding intersecting effects economic limitations, stigma, misinformation and lack of support often described moral hesitation and fear of health consequences, and exercised reproductive choice in a context of constraint. Nevertheless, reproductive agency emerged as something negotiated within personal experiences and the social context rather than an absolute pre‐determined by intersecting circumstances. Previous research studies have also pointed to the contextual nature of agency, taking into consideration how agency is shaped by identity, emotions, bodily experiences, and negotiation with social expectations, which are, in turn, very different across contexts and people (Cense 2019). The narratives also revealed an important distinction between individual and collective agency: although some excerpts framed SMA as a solitary do‐it‐yourself practice, others emphasised the role of feminist accompaniment organisations in offering a framework of solidarity that contrasted with dominant patriarchal and conservative social discourses, which is in line with previous research studies in the country (Burton and Peralta 2021).

Access to information about SRHR emerged as an important source of self‐determination, enabling individuals to exercise autonomy even in a constrained environment. In tandem, lack of information on how to obtain and or to use the pills was perceived as limiting their ability to act. Previous research studies have highlighted the key role of knowledge and information on SRHR for positive SMA experiences (Keefe‐Oates et al. 2025; World Health Organization 2022; Luigi‐Bravo et al. 2023) and demonstrated how a lack of information on abortion poses significant obstacles for women, resulting in delays that restrict their options and choice and may lead to unsafe abortion (Hinson et al. 2022). In Argentina, women still face barriers in accessing information about abortion due to the lack of easily accessible public information (Tiseyra et al. 2022; Vila Ortiz et al. 2024). Importantly, the politicisation of information access must be acknowledged. When public information is limited and/or misinformation is circulating—as is often the case in contexts with conservative governments or strong anti‐abortion movements—access becomes unequal: those with digital literacy, time, social networks and economic resources are better positioned to seek out and interpret information than those without such opportunities (Pagoto et al. 2023). In our study, data contributing to the *empowerment* narrative came from participants who had the economic and symbolic resources to access and interpret SRHR information and/or the relational factors to contact feminist accompaniment groups, enabling them to make informed decisions. For others with fewer resources and support, information was not as easily obtainable, demonstrating how unequal access intersects with broader structural constraints, a finding echoed by previous research studies (Ba et al. 2023; Ishola et al. 2021; Oberman 2018). In light of these findings, both health and political systems must go beyond simply ensuring availability of SRHR information towards addressing the inequities that shape who can access and act on such information.

Although SMA is a deeply personal and private experience for some women, it is not necessarily something people want to go through alone. Importantly, the type of support valued varied widely among participants: some would have liked the support of healthcare providers as they valued clinical expertise, whereas others preferred the presence of trusted friends or family members, or the accompaniment of women's organisations. Recent research studies support this finding, highlighting the importance of support in the abortion process (Keefe‐Oates et al. 2025; Rossier et al. 2021; Chor et al. 2019; Wilson‐Lowe et al. 2024). Hoggart et al. (2024) emphasised the importance of social connectedness as a critical factor in enabling a SMA positive experience. These differences highlight the need for a person‐centred approach to SMA support that is flexible and responsive to individual preferences. This suggests that support should be framed not as a gatekeeping mechanism but as a means of empowering and accompanying individuals through their chosen path (World Health Organization 2022).

Our findings highlight the importance of support systems that make space for the complexities of SMA, including physical pain during the process, doubt and uncertainty, not intending to eradicate them, but to provide tools for coping with them. Addressing these irreducible dimensions may be a valuable lesson for those who accompany abortion processes, whether in healthcare or accompaniment organisations. Even individuals with strong relational networks and confidence in their decision can find the experience more demanding than expected (Purcell et al. 2025). In this line, Siegel (2020) argues that discourses that tend to ‘normalise’ abortion as a routine practice or something that will necessarily become ‘easy’ under favourable conditions—often emerging from feminist efforts to counteract antiabortion narratives that frame abortion as traumatic—can create new normativities around abortion experiences, and may discourage conversations around difficult aspects of the practice. This has also been pointed out in a recent research study in Argentina (Vila Ortiz and Guaglianone 2025). These insights reinforce the need for support systems, whether formal or informal, to be attuned to the lived experiences of abortion seekers (Berer 2020) and to prioritise responsiveness to individual needs.

Our results call for more responsive and person‐centred public health strategies regarding SMA that are in line with the needs, preferences and lived realities of those seeking abortion care. For some abortion seekers, the formal system can offer a sense of safety and legitimacy. Others opt to be accompanied by other women in the community, as pointed out by studies in Latin America (Luchetti et al. 2024; Keefe‐Oates et al. 2025). Others may choose to manage the process in the privacy of their close sphere, as illustrated by some of the women in this study. This finding emphasises the need for health systems to improve their services, particularly in terms of emotional support and evidence‐based information. Crucially, this includes recognising that SMA is not only a response to legal restrictions but also a valid choice in contexts where abortion is legal. A truly person‐centred approach means offering individuals the option to manage their abortion in the way that is most aligned with their values, needs and circumstances, whether that involves clinical care, community accompaniment or privacy (Afulani et al. 2023). This flexibility is critical to enhancing reproductive agency. However, building person‐centred and flexible models of care requires an enabling social and legal environment and investments in health systems, both formal and community‐based. Considering the evolving social and institutional landscape in Argentina, this includes consideration of the conditions under which abortion care models can be sustained. In a context where the current national administration signals opposition to SRHR, policy priorities and budget allocations may undermine the expansion of public services. At the same time, feminist accompaniment collectives, inherently voluntary, are central actors in ensuring access and support, yet are often limited in scope because of several factors including funding.

Our study has several strengths and limitations. On the one hand, participants were self‐selected women who, independent of their experience, were willing to share their story of SMA, and most of whom were well‐educated women at the time of the interview. This means that many other experiences of SMA might not have been captured in our study. On the other hand, our recruitment strategy allowed us to access hidden stories about SMA that would otherwise have remained unknown. Although not looking for geographical representativeness, it is worth noting that participants came from four out of the 24 jurisdictions of the country. Therefore, our findings may not be transferable to other regions in the country, though they provide useful insight for national abortion policies.

## 6 Conclusion

Our analysis revealed that reproductive agency emerges in diverse and context‐dependent ways, ranging from decisive action to more constrained or implicit expressions of choice. Our findings highlight the importance of designing public health strategies that are person‐centred and that promote equitable access to abortion information and care. Whether SMA is pursued in response to legal restrictions or as a preferred method in legally permissive settings, it should be supported with flexible and responsive models of care. Recognising the emotional and physical dimensions of SMA, and the diverse forms of support people value, is essential to advancing agency. Acknowledging this complexity is critical to building models of abortion care that respect individual experiences, needs and preferences and enhance reproductive agency.

## Author Contributions

**Mercedes Vila Ortiz:** conceptualization, investigation, formal analysis, writing – original draft. **Fiorella Guaglianone:** formal analysis, writing – original draft. **Antonella Lavelanet:** methodology, validation, writing – review and editing. **Vanessa Brisuela:** validation, writing – review and editing. **Anna Thorson:** validation, writing – review and editing. **Anna Kågesten:** validation, writing – review and editing. **Mariana Romero:** methodology, formal analysis, writing – review and editing. **Amanda Cleeve:** conceptualization, methodology, supervision, formal analysis, project administration, writing – review and editing.

## Funding

This project was funded by the Swedish Research Council (2020‐02820) and (2013‐02289), and the Strategic Research Area Health Care Science, Karolinska Institutet (2021‐2023). The project was technically supported by the UNDP‐UNFPA‐UNICEF‐WHO‐World Bank Special Programme of Research, Development and Research Training in Human Reproduction (HRP), a cosponsored programme executed by the World Health Organization (WHO) (Project ID A66017).

## Ethics Statement

The project received ethical approvals from the WHO Ethics Review Commission [A66017], Investigación y Reflexión Bioética (www.irbioetica.com) and RESPIRE, Argentina. This study was submitted to the Swedish Ethics Review Authority [2023‐03332401] to ensure alignment with ethical standards although they do not formally approve studies conducted outside Sweden.

## Consent

Participants who agreed to participate in the study gave electronic consent and then verbal consent prior to the start of the recorded interview.

## Conflicts of Interest

The authors declare no conflicts of interest. AL and VB are staff members, and AT a previous staff member, in the UNFPA‐UNICEF‐WHO‐World Bank Special Programme of Research, Development and Research Training in Human Reproduction (HRP), a cosponsored programme executed by the World Health Organization (WHO). AT is a staff member in the UNICEF/UNDP/World Bank/WHO Special Programme for Research and Training in Tropical Diseases (TDR), a special program of the WHO. The named authors alone are responsible for the views expressed in this publication and do not necessarily represent the decisions or the policies of the HRP or TDR or WHO.

## Supporting information


Supporting Information S1

## Data Availability

Research data are not shared.

## Endnotes

^1^
“*Marea Verde” in Spanish, name of the feminist and social movement advocating for and supporting abortion legalisation Argentina.*

## References

[shil70181-bib-0001] Afulani, P. A. , M. K. Nakphong , and M. Sudhinaraset . 2023. “Person‐Centred Sexual and Reproductive Health: A Call for Standardized Measurement.” Health Expectations: An International Journal of Public Participation in Health Care and Health Policy 26, no. 4: 1384–1390. 10.1111/hex.13781. 37232021 PMC10349248

[shil70181-bib-0002] Atienzo, E. E. , B. Grosso , R. Zurbriggen , et al. 2023. “Characterizing Two Models for Abortion Care in Argentina Pre‐Law 27.610: 2016–2019.” Revista de Saúde Pública 57, no. 1: 36. 10.11606/s1518-8787.2023057004993. 37436261

[shil70181-bib-0003] Ba, D. M. , Y. Zhang , O. Pasha‐Razzak , et al. 2023. “Factors Associated With Pregnancy Termination in Women of Childbearing Age in 36 Low‐and Middle‐Income Countries.” PLOS Global Public Health 3, no. 2: e0001509. 10.1371/journal.pgph.0001509. 36963033 PMC10021843

[shil70181-bib-0004] Berer, M. 2020. “Reconceptualizing Safe Abortion and Abortion Services in the Age of Abortion Pills: A Discussion Paper.” Best Practice & Research Clinical Obstetrics & Gynaecology 63: 45–55. 10.1016/j.bpobgyn.2019.07.012. 31494046

[shil70181-bib-0005] Burton, J. , and G. T. Peralta . 2021. “Un Aborto feminista es un aborto cuidado: Prácticas de cuidado en el socorrismo patagónico.” Revista Estudos Feministas 29, no. 2: e70809. 10.1590/1806-9584-2021v29n270809.

[shil70181-bib-0006] Bury, L. , S. Aliaga Bruch , X. Machicao Barbery , and F. Garcia Pimentel . 2012. “Hidden Realities: What Women Do When They Want to Terminate An Unwanted Pregnancy in Bolivia.”Supplement International Journal of Gynecology & Obstetrics 118, no. Suppl 1: S4–S9. 10.1016/j.ijgo.2012.05.003. 22840269

[shil70181-bib-0007] Cense, M. 2019. “Rethinking Sexual Agency: Proposing a Multicomponent Model Based on Young People’s Life Stories.” Sex Education 19, no. 3: 247–262. 10.1080/14681811.2018.1535968.

[shil70181-bib-0008] Chor, J. , M. Tusken , D. Young , P. Lyman , and M. Gilliam . 2019. “Factors Shaping Women's Pre‐Abortion Communication With Members of Their Social Network.” Journal of Community Health 44, no. 2: 265–271. 10.1007/s10900-018-0582-1. 30306448 PMC6414232

[shil70181-bib-0009] Clandinin, D. J. , and F. M. Connelly . 2000. Narrative Inquiry: Experience and Story in Qualitative Research. Jossey‐Bass Publishers.

[shil70181-bib-0051] Cleeve, A. , X. Lu , M. Stein , et al. 2026. “Prevalence Estimation of Self‐Managed Abortion in Argentina: A Web‐Based Respondent‐Driven Sampling Study.” BMJ Sexual & Reproductive Health. 10.1136/bmjsrh-2025-203035. 41513447

[shil70181-bib-0010] Congreso de la Nación Argentina . 2021. Ley 27.610: Acceso a la interrupción voluntaria del embarazo y a la atención postaborto. Boletín Oficial de la República Argentina. https://www.argentina.gob.ar/normativa/nacional/ley‐27610‐346231.

[shil70181-bib-0011] Edmeades, J. , L. Hinson , M. Sebany , and L. Murithi . 2018. A Conceptual Framework for Reproductive Empowerment: Empowering Individuals and Couples to Improve Their Health. International Center for Research on Women.

[shil70181-bib-0012] Erdman, J. N. , K. Jelinska , and S. Yanow . 2018. “Understandings of Self‐Managed Abortion as Health Inequity, Harm Reduction and Social Change.” Reproductive Health Matters 26, no. 54: 13–19. 10.1080/09688080.2018.1511769. 30231807

[shil70181-bib-0013] Hinson, L. , A. M. Bhatti , M. Sebany , et al. 2022. “How, When and Where? A Systematic Review on Abortion Decision Making in Legally Restricted Settings in Sub‐Saharan Africa, Latin America, and the Caribbean.” BMC Women's Health 22, no. 1: 415. 10.1186/s12905-022-01962-0. 36217197 PMC9552475

[shil70181-bib-0014] Hoggart, L. , C. Purcell , F. Bloomer , V. Newton , and A. Oluseye . 2024. “Social Connectedness and Supported Self‐Management of Early Medication Abortion in the UK: Experiences From the COVID‐19 Pandemic and Learning for the Future.” Culture, Health and Sexuality 26, no. 7: 855–870. 10.1080/13691058.2023.2258189. 37830180

[shil70181-bib-0015] Hyatt, E. G. , J. L. M. McCoyd , and M. F. Diaz . 2022. “From Abortion Rights to Reproductive Justice: A Call to Action.” Affilia 37, no. 2: 194–203. 10.1177/08861099221077153.

[shil70181-bib-0016] Ishola, F. , U. V. Ukah , B. Y. Alli , and A. Nandi . 2021. “Impact of Abortion Law Reforms on Health Services and Health Outcomes in Low‐ and Middle‐Income Countries: A Systematic Review.” Health Policy and Planning 36, no. 9: 1483–1498. 10.1093/heapol/czab069. 34133729

[shil70181-bib-0017] Jacobson, L. E. , and C. Gerdts . 2025. “Self‐Managed Medication Abortion: History, Evidence, Models of Care, and Policy Considerations.” American Journal of Public Health 115, no. 10: e1–e8. 10.2105/AJPH.2025.308133. PMC12424507 40532124

[shil70181-bib-0018] Keefe‐Oates, B. , S. Filippa , E. Janiak , et al. 2025. “Seeking Abortion Accompaniment: Experiences and Self‐Managed Abortion Preferences of Hotline Callers After Abortion Legalisation in Argentina.” BMJ Sexual & Reproductive Health 51, no. 2: 152–158. 10.1136/bmjsrh-2023-202209. 38889960

[shil70181-bib-0019] Keefe‐Oates, B. , C. G. Tejada , R. Zurbriggen , B. Grosso , and C. Gerdts . 2022. “Abortion Beyond 13 Weeks in Argentina: Healthcare Seeking Experiences During Self‐Managed Abortion Accompanied by the Socorristas En Red.” Reproductive Health 19, no. 1: 185. 10.1186/s12978-022-01488-6. 36028868 PMC9419329

[shil70181-bib-0020] Kristianingrum, I. A. , S. Nmezi , R. Zurbriggen , C. Gerdts , R. Jayaweera , and H. Moseson . 2022. “Overcoming Challenges in Research on Self‐Managed Medication Abortion: Lessons From a Collaborative Activist–Researcher Partnership.” Sexual and Reproductive Health Matters 30, no. 1: 2077282. 10.1080/26410397.2022.2077282. 35695259 PMC9225767

[shil70181-bib-0021] Lorey, I. 2015. State of Insecurity: Government of the Precarious. Verso.

[shil70181-bib-0022] Luchetti, G. , V. Albardonedo , and M. V. Alfonso . 2024. “Socorristas en red: Soporte comunitario a la autogestión del aborto en Argentina [Women's Rescuers Network: Community Support for self‐managed Abortion in Argentina].” Salud colectiva 20: e4810. 10.18294/sc.2024.4810. 38992339 PMC11822894

[shil70181-bib-0023] Luigi‐Bravo, G. , A. Maria Ramirez , C. Gerdts , and R. Gill . 2023. “Lessons Learned From Developing and Implementing Digital Health Tools for Self‐Managed Abortion and Sexual and Reproductive Healthcare in Canada, the United States, and Venezuela.” Sexual and reproductive health matters 31, no. 4: 2266305. 10.1080/26410397.2023.2266305. 37870150 PMC10595388

[shil70181-bib-0024] Madeiro, A. P. , and D. Diniz . 2015. “Induced Abortion Among Brazilian Female Sex Workers: A Qualitative Study.” Ciência & Saúde Coletiva 20, no. 2: 587–593. 10.1590/1413-81232015202.11202014. 25715152

[shil70181-bib-0025] Moseson, H. , R. Jayaweera , I. Egwuatu , et al. 2022. “Effectiveness of Self‐Managed Medication Abortion With Accompaniment Support in Argentina and Nigeria (SAFE): A Prospective, Observational Cohort Study and Non‐Inferiority Analysis With Historical Controls.” Lancet Global Health 10, no. 1: e105–e113. 10.1016/s2214-109x(21)00461-7. 34801131 PMC9359894

[shil70181-bib-0026] National Supreme Court of Justice of Argentina . 2012. “F.A.L. s/Medida Autosatisfactiva.” Fallos 335: 197. https://www.saij.gob.ar/corte‐suprema‐justicia‐nacion‐federal‐ciudad‐autonoma‐buenos‐aires‐‐medida‐autosatisfactiva‐fa12000021‐2012‐03‐13/123456789‐120‐0002‐1ots‐eupmocsollaf.

[shil70181-bib-0027] Oberman, M. 2018. “Motherhood, Abortion, and the Medicalization of Poverty.” Journal of Law Medicine & Ethics: A journal of the American Society of Law, Medicine & Ethics 46, no. 3: 665–671. 10.1177/1073110518804221. 30336087

[shil70181-bib-0028] Olmos‐Vega, F. M. , R. E. Stalmeijer , L. Varpio , and R. Kahlke . 2022. “A Practical Guide to Reflexivity in Qualitative Research: AMEE Guide No. 149.” Medical Teacher 45, no. 3: 241–251. 10.1080/0142159X.2022.2057287. 35389310

[shil70181-bib-0029] Olwanda, E. , K. Opondo , D. Oluoch , et al. 2024. “Women's Autonomy and Maternal Health Decision Making in Kenya: Implications for Service Delivery Reform ‐ A Qualitative Study.” BMC Women's Health 24, no. 1: 181. 10.1186/s12905-024-02965-9. 38504293 PMC10949706

[shil70181-bib-0030] Ortner, S. B. 2006. Anthropology and Social Theory: Culture, Power, and the Acting Subject. Duke University Press. 10.1215/9780822388456.

[shil70181-bib-0031] Pagoto, S. L. , L. Palmer , and N. Horwitz‐Willis . 2023. “The next Infodemic: Abortion Misinformation.” Journal of Medical Internet Research 25: e42582. 10.2196/42582. 37140975 PMC10196890

[shil70181-bib-0032] Purcell, C. , V. L. Newton , F. Bloomer , and L. Hoggart . 2025. “Foregrounding Pain in Self‐Managed Early Medication Abortion: A Qualitative Study.” BMJ Sexual & Reproductive Health 51, no. 1: 3–8. 10.1136/bmjsrh-2023-202198. 38429082

[shil70181-bib-0033] Ramón Michel, A. , and S. Ariza Navarrete . 2018. La legalidad del aborto en la Argentina (Serie Documentos REDAAS N° 9). REDAAS, ELA, CEDES. https://redaas.org.ar/wp‐content/uploads/La‐legalidad‐del‐aborto‐en‐la‐Argentina.pdf.

[shil70181-bib-0034] Ramos, S. , B. Keefe‐Oates , M. Romero , et al. 2023. “Step by Step in Argentina: Putting Abortion Rights Into Practice.” International Journal of Women's Health 15: 1003–1015. 10.2147/IJWH.S412975. PMC10349583 37455681

[shil70181-bib-0035] Rebughini, P. 2023. “Agency in Intersectionality: Towards a Method for Studying the Situatedness of Action.” European Journal of Cultural and Political Sociology 10, no. 1: 1–20. 10.1080/23254823.2023.2174963.

[shil70181-bib-0036] Riessman, C. K. 1993. Narrative Analysis. Sage Publications.

[shil70181-bib-0037] Riley, T. , and P. Hawe . 2005. “Researching Practice: The Methodological Case for Narrative Inquiry.” Health Education Research 20, no. 2: 226–236. 10.1093/her/cyg122. 15479707

[shil70181-bib-0038] Romero, M. , B. Keefe‐Oates , M. Krause , A. Ramón Michel , and S. Ramos . 2024. Reporte anual 2023: Logros de la política de acceso al aborto y amenazas actuales. Centro de Estudios de Estado y Sociedad (CEDES). https://proyectomirar.org.ar/wp‐content/uploads/PM_reporte‐anual‐2023.pdf.

[shil70181-bib-0039] Romero, M. , and A. Ramón Michel . 2022. “The Shift From Criminalization to Legalization of Abortion in Argentina.” JAMA 328, no. 17: 1699–1700. 10.1001/jama.2022.18971. 36318122

[shil70181-bib-0040] Rossier, C. , A. Marchin , C. Kim , and B. Granata . 2021. “Disclosure to Social Network Members Among Abortion‐Seeking Women in Low‐ and Middle‐Income Countries With Restrictive Access: A Systematic Review.” Reproductive Health 18, no. 1: 114. 10.1186/s12978-021-01165-0. 34098958 PMC8186048

[shil70181-bib-0041] Siegel, D. 2020. “Medicalization and Naturalization: Understanding Abortion as a Naturecultural Phenomenon.” Catalyst: Feminism, Theory, Technoscience 6, no. 2. 10.28968/cftt.v6i2.34030.

[shil70181-bib-0042] Stapff, C. , L. G. Garbero , R. G. Ponce de León , L. Briozzo , A. Lavelanet , and M. Narasimhan . 2025. “Self‐Care Interventions for Legal and Safe Abortions: Lessons Learned From a Woman‐Centered Approach to Sexual and Reproductive Healthcare in Uruguay.” Lancet Regional Health Americas 42: 100981. 10.1016/j.lana.2024.100981. 39877307 PMC11773253

[shil70181-bib-0043] Szulik, D. E. , and N. Zamberlin . 2020. “La legalidad oculta: Percepciones de estigma en los recorridos de mujeres que descubren y acceden a la interrupción legal del embarazo por causal salud.” Sexualidad, Salud y Sociedad ‐ Revista Latinoamericana 34: 46–67. 10.1590/1984-6487.sess.2020.34.04.a.

[shil70181-bib-0044] Tiseyra, M. V. , M. Vila Ortiz , M. Romero , E. Abalos , and S. Ramos . 2022. “Barreras de acceso al aborto legal en el sistema público de salud de dos jurisdicciones de Argentina: Rosario y Ciudad Autónoma de Buenos Aires, 2019‐2020.” Salud Colectiva 18: e4059. 10.18294/sc.2022.4059. 36520496

[shil70181-bib-0045] Vila Ortiz, M. , and F. Guaglianone . 2025. “¿Puede el aborto ser una práctica reproductivista? Tensiones entre reproductivismo y emancipación en algunos relatos de interrupciones de embarazos.” Temas Y Debates, no. 49: 39–54. 10.35305/tyd.vi49.687.

[shil70181-bib-0046] Vila Ortiz, M. , M. V. Tiseyra , M. Romero , et al. 2024. “Trusted Networks: A Study of Communication Flow and Access to Abortion Information in Argentina.” Culture, Health and Sexuality 27, no. 10: 1–16. 10.1080/13691058.2024.2408345. 39351919

[shil70181-bib-0047] Wilson‐Lowe, R. V. , C. Purcell , R. Lewis , and R. Eastham . 2024. “Seeking Support for Abortion Online: A Qualitative Study of Women’s Experiences.” BMJ Sexual & Reproductive Health 50, no. 2: 172–177. 10.1136/bmjsrh-2023-202083. 38336468 PMC11287619

[shil70181-bib-0048] World Health Organization . 2022. Abortion Care Guideline. World Health Organization. https://www.who.int/publications/i/item/9789240039483.

[shil70181-bib-0049] Zamberlin, N. , M. Romero , and S. Ramos . 2012. “Latin American Women's Experiences With Medical Abortion in Settings Where Abortion is Legally Restricted.” Reproductive Health 9, no. 1: 34. 10.1186/1742-4755-9-34. 23259660 PMC3557184

[shil70181-bib-0050] Zurbriggen, R. , B. Keefe‐Oates , and C. Gerdts . 2018. “Accompaniment of Second‐Trimester Abortions: The Model of the Feminist Socorrista Network of Argentina.” Contraception 97, no. 2: 108–115. 10.1016/j.contraception.2017.07.170. 28801052

