# Supplementary material for: ‘Come on, Give Me the Pills Now’: A Narrative Analysis of Reproductive Agency in Self‐Managed Abortion in Argentina
Source: Sociol Health Illn. 2026 Apr 8;48(4):e70181. doi: 10.1111/1467-9566.70181 (PMC13059537; doi:10.1111/1467-9566.70181)
Supplement: Supplementary file 1 — Supporting Information S1 [file SHIL-48-0-s001.docx]

**Annex 1**

**Interview guide**

Date:

Interview number:

Interviewer initials:

_________________________________________________________________________

*Thank you for agreeing to participate in this interview. You participated in our online survey and indicated that you have experience of abortion with pills at home. Is this information correct?*

*With this interview, we hope to get a deeper understanding of your abortion experience.*

Could you tell me, in your own words, about your abortion experience beginning from the moment you realised you were pregnant?

*Probes:*

- *Abortion decision-making (including influence from other ppl, coercion etc.)*
- *Why abortion with pills at home?*
- *What were your main concerns?*
- *How did you access the pills?*
- *Support for what, from where, whom, how?*
- *How did you manage your doubts, uncertainties, unexpected problems?*
- *Post abortion contraceptives?*

If you were able to go back and change anything about this experience, what would it be?

*Explore preferences and needs*

- *information, sources of information, sources of medications and instructions*
- *Support, purpose, type, mode, timing, etc.*
- *Manage of the doubts, uncertainties, unexpected problems*
- *Access to other SRH services (contraception, STI/HIV testing, services for GBV)*

Women and girls experience abortion very differently; some describe feeling very strong and in control and others describe feeling powerless. Could you tell me about how you felt during and after this abortion?

*Probes:*

- *Did your feelings change along the process? How?*
- *What made you feel this/these ways?*
- *Did you feel you were able to make your own choices and/or follow your preferences? Voice your opinions?*
- *Did this experience impact your confidence in your abilities to deal with your reproductive health decisions or other realms of your life? How?*
- *Impact on self-advocacy? Self-determination? Sense of control? How?*

Is there anything else that you would like to add?

Thank you for your participation!

**Annex 2**

**Summary of the mapping process**

| **MAPPING PROCESS** | **Type of coding** | **What was coded** | | **Examples** |
| --- | --- | --- | --- | --- |
|  | Inductively coded | Content of each story | | Pathway respondents followed to get the pills, where, and when they carried out the SMA, with WHOM |
|  | Deductively coded | Agency | Voice | Whether and how respondents were able to express preferences and needs |
|  |  |  | Choice | Whether respondents narrated if and how they were able to make autonomous decisions |
|  |  |  | Power | Power relations and struggles, including resistance, influence, solidarity, and other forms of interaction |
|  |  | Circumstances that shaped agency | Personal | Knowledge about sexual and reproductive health, including SMA |
|  |  |  | Relational | Support during the SMA process |
|  |  |  | Structural | Economic, social, and symbolic resources to access abortion medication and healthcare services |
|  |  | Dimensions of narrative inquiry | Temporality | How the abortion was narrated concerning the time it took place, i.e., ' at that time, nobody talked about abortion’ |
|  |  |  | Sociality | How social and cultural values and discourses around abortion appeared in the story, i.e., abortion as taboo versus openness to talk about it |
|  |  |  | Spatiality | The context in which the story was situated, especially concerning the availability of abortion care services and the legal framework |
